# Recombinant Phage Elicits Protective Immune Response against Systemic *S. globosa* Infection in Mouse Model

**DOI:** 10.1038/srep42024

**Published:** 2017-02-06

**Authors:** Feng Chen, Rihua Jiang, Yicun Wang, Mingji Zhu, Xu Zhang, Shuai Dong, Hongxi Shi, Li Wang

**Affiliations:** 1 Dermatology Department, China-Japan Union Hospital of Jilin University, 126Xiantai Street, Changchun, Jilin Province 130033, People’s Republic of China; 2 Jilin Provincial Key Laboratory on Molecular and Chemical Genetic, the Second Hospital of Jilin University, 218 Ziqiang Street, Changchun, Jilin Province 130041, People’s Republic of China; 3 Department of Hang Surgery, Second Hospital of Qinhuangdao, Hebei 066600, People’s Republic of China; 4 Institute of Cytology and Genetics, School of Life Sciences, Northeast Normal University, 5268 Renmin Street, Changchun, Jilin Province 130024, People’s Republic of China

## Abstract

*Sporothrix globosa* is a type of fungus that typically infects immunocompromised patients. Its prevention continues to pose a challenge. A 70-KDa glycoprotein (Gp70) of *Sporothrix* has been previously reported to protect host against infection from this fungus. Here, we displayed an epitope peptide (kpvqhalltplgldr) of Gp70 on the major coat protein (pIII), and investigated its efficiency as a vaccine for preventing *S. globosa* infection. The recombinant phage and the heat-killed *S. globosa* were used to immunize mice separately. In this study, we evaluated the humoral and cellular immune responses in the mice and demonstrated that recombinant phage could induce mice to produce a stronger immune response and generate antibodies to inhibit *S. globosa* infection. Furthermore, immunization with recombinant phage could increase the survival rate of *S. globosa* infection in mice. All these results together indicated that recombinant phages displaying kpvqhalltplgldr are a potential vaccine candidate against *S. globosa* infection.

Sporotrichosis is an acute or chronic subcutaneous mycosis caused by the *Sporothrix* complex. The global spread of sporotrichosis infection has been increasing in recent years^1^. The *Sporothrix* complex comprises at least six sibling phylogenetic species, including *S. pallida, S. brasiliensis, S. globosa, S. luriei, S. mexicana,* and *S. schenckii*^2^,^3^. To our knowledge, S*. globosa* is the most common pathogenic species in Northeast China, irrespective of clinical symptoms or regions where it is isolated^4^. Severe clinical forms of sporotrichosis mainly occur in immunocompromised patients, especially in patients infected with human immunodeficiency virus (HIV). In addition, disseminated cutaneous sporotrichosis has been reported in immunocompetent individuals^5^,^6^. Antifungal drugs are commonly used in clinical practice, but they are associated with considerable severe adverse effects such as vomiting, diarrhea, headache, abdominal pain, hypersensitivity, liver dysfunction, and fungal resistance^3^,^7^,^8^.

Humoral and cellular immunities are the most important host defense mechanisms against fungal infections. Therapeutic monoclonal antibodies against *S. schenckii* are included in the most active fields of laboratory^9^. A growing number of antifungal vaccine candidates have been assessed for their immunogenicity, safety, and ability to confer protection against fungal pathogens^10^,^11^,^12^,^13^,^14^,^15^. A previous study on a mouse model revealed that immunization of mice with *Sporothrix spp.* cell wall proteins or whole cells could elicit protective immune responses against subsequent infection of *Sporothrix* spp^10^,^12^. However, no vaccine against *Sporothrix spp.* has yet been widely applied in the clinical setting.

Gp70 is a 70-kDa glycoprotein that has been described as a major adhesion factor on the cell surface of *Sporothrix*. It is a cross-immunogenic protein associated with virulence in fungi that undergoes different post-translational modifications and is present as isoforms and glycoforms in the *S. schenckii* complex proteomes^16^,^17^. Monoclonal antibodies (mAbs) against Gp70 have been demonstrated to induce strong protection against *Sporothrix* fungi^10^. Epitopes (avyvtsntehnsvvaipiar, gptntvshvffsgdqetvfttvk, tvipgqdatcwvaicpathtafvtdir, kpvqhalltplgldr) on Gp70 were identified by application of epitope-finding algorithm (GenScript, Antigen Design Tool, Optimum Antigen).

Phages displaying peptides can induce humoral and cell-mediated immune responses without the need for an adjuvant. Therefore, phages can be an effective delivery system and be useful for the design of vaccines without compromising their safety or tolerability. In our preliminary experiments, phage displaying the peptide “kpvqhalltplgldr” was found to effectively improve the survival time of the mouse. Therefore, in this study, we aimed to display the peptide kpvqhalltplgldr (referred to as KR) on phages and to study the efficacy of this recombinant phage as a vaccine. The recombinant phage represents a potential novel vaccine candidate without the need for an adjuvant against *S. globosa*.

## Results

### Production of recombinant phage

**Figure 1:**
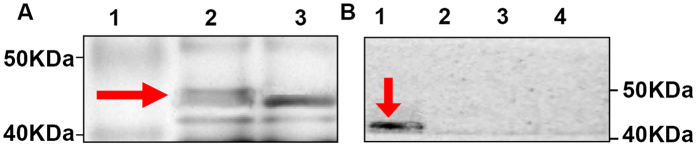
Expression and antigenicity of phage-displayed kpvqhalltplgldr peptide. (**A**) SDS-PAGE (20%) of recombinant phage. Lane 1, protein markers; lane 2, recombinant phage displaying the kpvqhalltplgldr peptide; lane 3, wild-type phage. (**B**) Western blot analysis of the recombinant and wild-type phages with infected mouse serum containing antibodies to a 70-kDa protein band or with the serum from a control mouse. Lane 1, recombinant phage was probed with infected mouse serum; Lane 2, wild-type phage was probed with infected mouse serum. Lane 3, recombinant phage was probed with normal serum. Lane 4, wild-type phage was probed with normal serum.

Expression of phage-KR was assessed using SDS-PAGE, and a 46-kDa protein band lagged the wild-type phage (Fig. 1A). Serum samples collected from infected mice reacted using the fusion protein band in recombinant phage (Fig. 1B). This demonstrated that the recombinant phage displayed the KR peptide on its surface.

### Antibody response against phages displaying the KR peptide in immunized mice

**Figure 2:**
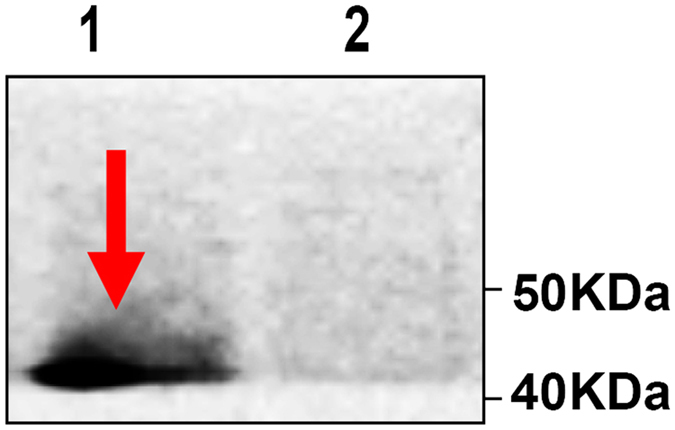
Immunization with recombinant phage induces kpvqhalltplgldr-specific humoral immunity in mice. Western blot analysis of sera from recombinant phage-immunized mice with recombinant phage (lane 1), wild-type phage (lane 2). Lanes 1, recombinant phage; Lane 2, wild-type phage.

**Figure 3:**
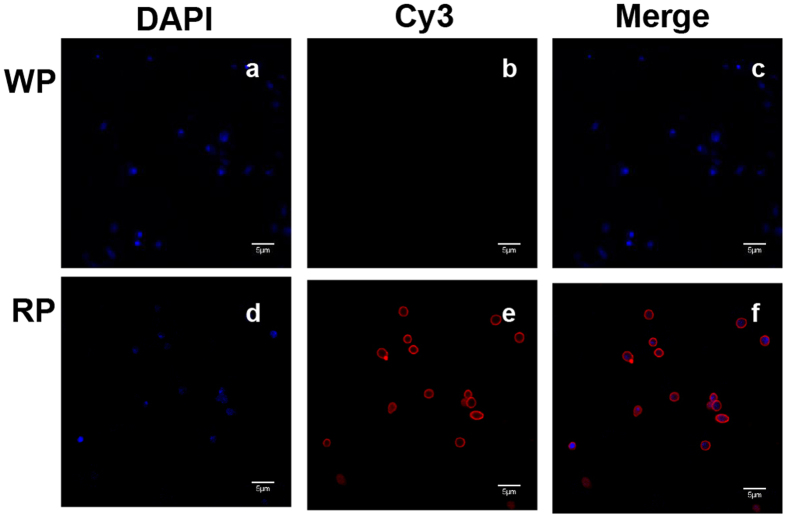
Immunofluorescence to assess the binding affinity of antibody containing sera with *S. globosa*. (**a**) *S. globosa* stained with DAPI (excitation: 358 nm, emission: 461 nm), (**b**) *S. globosa* incubated with serum containing anti-WP and stained with goat anti-mouse IgG conjugated with Cy3 (excitation: 550 nm, emission: 570 nm), (**c**) Merged image of (**a**) and (**b**). (**d**) *S. globosa* stained with DAPI (excitation: 358 nm, emission: 461 nm), (**e**) *S. globosa* incubated with sera containing anti-RP (recombinant phage displaying peptide-kpvqhalltplgldr) and stained by goat anti-mouse IgG conjugated with Cy3 (excitation: 550 nm, emission: 570 nm), (**f**) merged image of (**d**) and (**e**). Fluorescence images illustrate binding of the serum antibodies collected from RP-immunized mice to *S. globosa*. RP: recombinant phage, WP: wild-type phage.

Our study showed that immunization with the recombinant phage displaying the kpvqhalltplgldr peptide (RP) could produce serum antibodies (Figs 1 and 2), which can bind to Gp70 (Fig. 3). Together, our work shows that the RP displaying the kpvqhalltplgldr peptide can serve a function similar to that of Gp70 in the treatment of *S. globosa* infection and has potential for use as a vaccine. The results of our study indicate that the kpvqhalltplgldr peptide displayed on the phage surface can mimic Gp70 and induce Gp70-specific antibody production in mice, which can in turn bind with Gp70 and treat the infection. Some studies have claimed that mice inoculated with monoclonal antibodies against Gp70 exhibited significant passive protection^10^,^18^. Similar to these findings, our results prove that treatment with anti-RP IgG conferred significant protection against *S. globosa* infection.

### Recombinant-phage induced Th1 and Th17 mixed response

**Figure 4:**
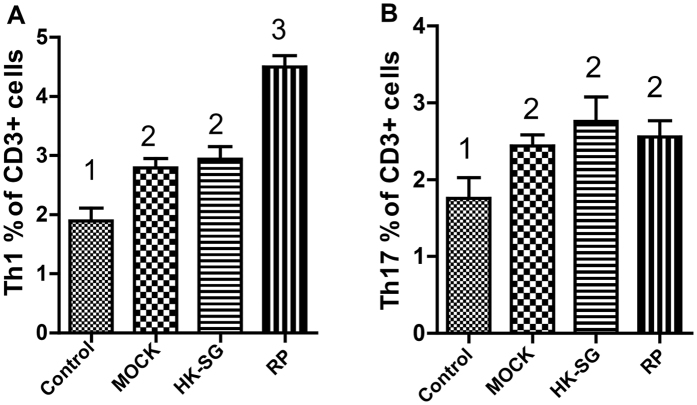
Using flow cytometry to analyze the populations of Th1 and Th17 cells. The percentages of Th1 cells of mice in the RP group were significantly higher than the corresponding values in the controls (mock and HK-SG groups) (P = 0.041) while the values did not significantly differ between the mock and HK-SG groups (**A**). The percentages of Th17 cells of mice in the RP, mock, and HK-SG groups were significantly higher than that of the control group (P = 0.013), while the values did not significantly differ between the RP, mock, and HK-SG groups (**B**). Values followed by different capital letters differ significantly among the Control, MOCK, HK-SG, and RP groups. RP: recombinant phage, MOCK: wild-type phage; HK-SG: heat-killed *S. globosa*.

We used flow cytometry analysis to test the immune skewing of the T cells (Fig. S1). The results revealed that percentages of Th1 cells among mice in the RP group were significantly higher than the corresponding percentage in the control groups (mock and HK-SG groups; *p* = 0.041), while there were no significant differences between the percentages of Th1 cells between the mock and HK-SG groups (Fig. 4A). The percentages of Th17 cells were significantly higher in the RP, mock, and HK-SG groups than in the control group (*p* = 0.013), although there were no significant differences in the values among the RP, mock, and HK-SG groups (Fig. 4B).

### Protection by recombinant phage against systemic S. globosa infection in BALB/c mice

**Figure 5:**
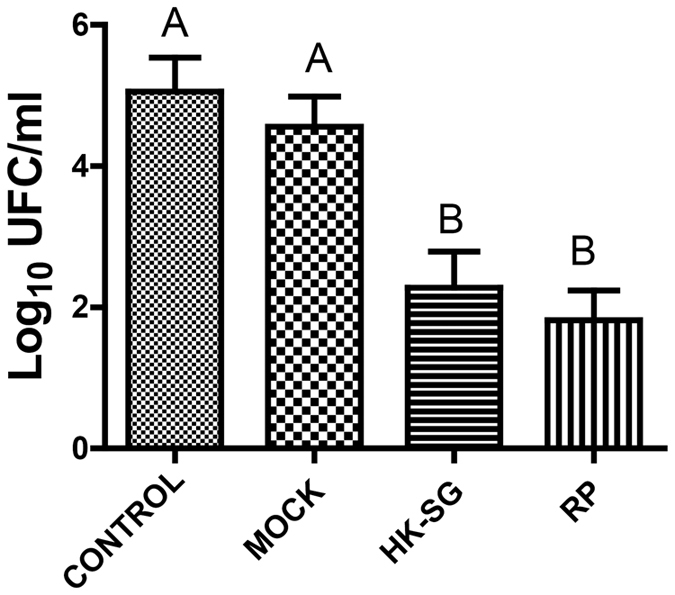
Log_10_ CFU in mouse kidneys. To determine whether immunization with hybrid phage could reduce the *S. globosa* levels in organs, the mice were sacrificed, and the log_10_ CFU was quantified in kidneys 7 days after the mice were immunized. There were significantly fewer log_10_ CFU in the organs of hybrid phage-immunized mice than in those that immunized with PBS (control group) and wild-type phage (mock group). There was no statistically significant difference in clearance from the kidney between the HK-SG-immunized group and recombinant phage-immunized group. Values followed by different capital letters differ significantly among the control, mock, HK-SG, and RP groups. RP: recombinant phage, MOCK: wild-type phage; HK-SG: heat-killed *S. globosa*.

**Figure 6:**
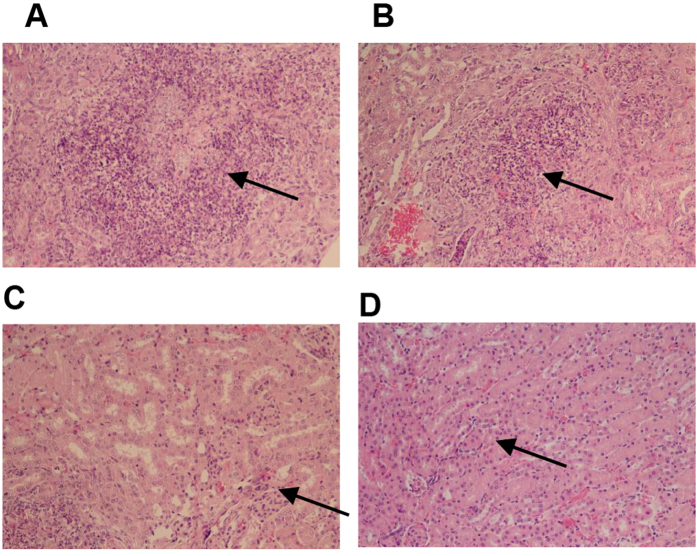
Representative micrographs of HE-stained kidneys of *S. globosa*-infected mice. Kidneys were removed from mice given intraperitoneal injections of PBS (100 μl/mouse), recombinant phage (25 μg/mouse), heat-killed *S. globosa* (10^8^ yeast cells/mouse), or wild-type phage (25 μg/mouse) four times at weekly intervals, and then intravenously infected with 2 × 10^7^ *S. globosa* yeast cells. (**A**) Kidney from a mouse injected with PBS. Magnification, 200 × . (**B**) Kidney from a mouse immunized with wild-type phage. Magnification, 200 × . (**C**) Kidney from a mouse immunized with heat-killed *S. globosa* (HK-SG). Magnification, 200 × . (**D**) Kidney from a mouse immunized with recombinant phage. Magnification, 200 × . Photomicrographs (**A**) and (**B**) exhibited show a greater number of inflammatory cells in kidneys than group HK-SG (**C**) and group RP (**D**). The arrows indicate infiltration of inflammatory cells.

**Figure 7:**
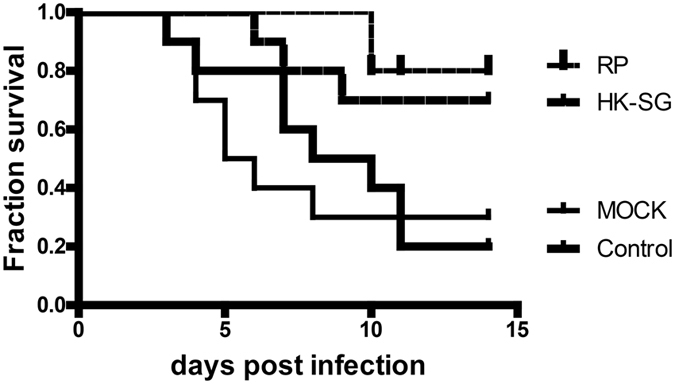
Survival time of the mice. Mice receiving recombinant phage (25 μg/mouse), wild-type phage (mock group; 25 μg/mouse), HK-SG (10^8^ yeast cells/mouse), and PBS (100 μg/mouse) four times at weekly intervals followed by intravenous challenge with 1 × 10^8^ viable *S. globosa*. 1 week after the last exposure to antigen. Statistical test at day 15: recombinant-phage-immunized group vs. PBS-injected group: *P* = 0.005; HK-SG-immunized group vs. PBS-injected group: *P* = 0.044; wild-type phage-immunized group vs. PBS-injected group: *P* = 0.79 (by log-rank test). RP: recombinant phage, MOCK: wild-type phage. HK-SG: heat-killed *S. globosa*.

Mice were sacrificed to determine whether immunization with recombinant phage could reduce the fungal load. The CFU was quantified in kidneys 7 days after infection, and the results were log_10_ transformed. The CFU was significantly lower in the organs of recombinant phage-immunized mice than those that received phosphate-buffered saline (PBS; Fig. 5). Regarding clearance from the kidney, there were no significant differences between the HK-SG-immunized and recombinant phage-immunized groups. Similar results were observed in the livers (Fig. S2). Histopathological examination showed that fewer inflammatory cells were seen in the kidney in the recombinant phage (Fig. 6D)- and HK-SG (Fig. 6C)-immunized mice than in the PBS (Fig. 6A)- and wild-type phage-immunized mice (Fig. 6B). Also, PBS (Fig. S3A) and wild-type phage-immunized (Fig. S3B) mice showed a greater number of inflammatory cells than recombinant phage (Fig. S3D) and HK-SG-immunized (Fig. S3C) mice. Taken together, these findings revealed that protective immunity to *S. globosa* could be induced by immunization with recombinant phage and HK-SG. Similar results were obtained in the replicates. Furthermore, recombinant phage was tested for its ability to protect animals from sublethal disseminated sporotrichosis. The procedure was established by intravenous inoculation of viable yeast cells (0.2 mL, 1 × l0^8^ cells/mouse) in BALB/c mice. Figure 7 shows the survival time of each group (n = 10) over 14 days following sublethal challenge. The highest survival rates (80%) were seen in mice that were immunized with recombinant phage. In contrast, only 20% of the mice injected with PBS survived. For the animals receiving HK-SG, a greater survival rate was observed at 14 days after infection (70% of the mice immunized with HK-SG were still alive). A significant increased survival rate was observed in recombinant phage-immunized mice compared to PBS-injected mice.

### Toxicity study

The toxicological assessment of recombinant phage was performed. Food intake and body weight gain were unaffected by immunization. All hematological parameters (red and white blood cell counts and platelet count) and clinical biochemistry parameters (aspartate aminotransferase, alanine aminotransferase, alkaline phosphatase, glucose, urea, creatinine, total protein, and albumin) showed values well within the biological range. We observed no differences between the treatment and control groups either at 24 h or 21 days after the first immunization (data not shown).

## Discussion

Currently, an increasing number of studies is focused on application of vaccine against fungal infection, as well as on the increasing morbidity and mortality of antifungal therapies. Whole-cell and protein vaccines can exert protective immunity against fungal infection by inducing humoral and cellular immune responses. However, thus far, few vaccines have been widely used in clinical practice due to their adverse effects. In our study, recombinant phages displaying kpvqhalltplgldr (referred to as phage-KR) were a potential vaccine candidate against *S. globosa* infections, as they were found to induce strong humoral and cellular immune responses in a mouse model in this study.

FDA has recently approved the safety of phage-based products as food additives. In our toxicity study, the addition of phage did not affect hematological parameters (red and white blood cell counts and platelet count) or the biochemical parameters (aspartate aminotransferase, alanine aminotransferase, alkaline phosphatase, glucose, urea, creatinine, total protein, and albumin).

The host defense against *S. globosa* infection includes humoral and cellular immunity. There is a series of evidence indicating that protective antibodies and CD4^+^ T helper 1 (Th1) and Th17 cells are key elements in eliciting immune responses against *S. schenckii* infection^6^,^10^,^19^,^20^,^21^. In our study, phage-KR effectively protected the host against *S. globosa* infection by inducing humoral and cellular immunity.

Thl responses are involved in activating macrophages by releasing interferon-gamma (IFN-γ), a strong macrophage activator^22^. IL-17 produced by Th17 can recruit neutrophils, which are involved in fungistatic and fungicidal responses. Absence of these responses is related to a higher lethality in experimental infections in mice^23^. As the key factors in controlling fungal infection, Th1 and Th17 are of great importance in the pathogenesis of sporotrichosis. However, clinical manifestations may vary based on the activation of immunity^24^. T cell activation can be triggered by different infectious agents such as *C. albicans*. Mice injected with phage-KR had high levels of IFN-γ + Th1 and IL-17 + Th17, indicating that the recombinant phage may induce a protective cell-mediated immune response against *S. globosa* infection by inducing the production of IFN-γ and IL-17.

It was previously shown^13^ that gp70 is a putative adhesion for fibronectin and laminin, and antibody against gp70. *S. schenckii* yeast cells are prevented from interacting with the subendothelial matrix by the immune system. Yeast cells that were opsonized with monoclonal antibodies against the 70-kDa protein were found to have a higher phagocytic index, indicating that the antibody treatment may have induced a protective cell-mediated immune response by producing IFN-γ. IFN-γ is considered to be the most important cytokine for conferring protection from sporotrichosis^25^,^26^. Murine serum infected with *S. globosa* was specifically recognized by phage-KR (Fig. 1), indicating that phage-KR could mimic the native KR peptide on the 70-kDa protein. Phages are also a powerful vaccine vector. The immunogenicity of epitopes is enhanced when displayed on phages. Murine antisera raised against recombinant phage reacts with the KR peptide (Figs 1 and 2). Results of the Western blot analysis using serum samples from the experimental mice were pre-incubated with wild-type phage. The results revealed that the specific antibody response to recombinant phages may be stimulated by the KR peptide rather than the phage components. Antibodies are natural products of the immune system and interact with other immune components. Protective antibodies may function via complement-mediated lysis, enhancement, inhibition of phagocytosis, Fc-mediated cytokine release, and direct antimicrobial effects^27^. The present study is the first to demonstrate antibodies against *S. globosa* induced by phage-KR, which may effectively protect mice from disseminated *S. globosa* infection.

In order to evaluate the protective effect of RP, initiative immunization was tested using *S. globosa*-infected mice. The number of CFU and inflammatory cells in the kidney were significantly lower in the phage KR-immunized mice prior to infection with *S. globosa*.

Inactivated microbes are a type of vaccine. Microorganisms can be inactivated by heat, radiation, and certain chemicals. The inactivated microbe will no longer cause illness, but can still be recognized by the immune system. Polio virus and Hepatitis A are the most common microbes used in the preparation of inactivated vaccines^7^. Inactivated vaccines are more effective than other vaccines over a shorter duration. Multiple vaccine boosters may improve the efficiency and sustainability, and may sometimes cause local swelling, pain, and fever^28^. The phage displaying epitope vaccine can be produced easily and cost effectively in a large quantity, because it is assembled in the cytoplasm of *E. coli* cells. The progeny phage is released by cell lysis. The particles are extremely robust and stable to harsh conditions. As the highly immunogenic synthetic vaccines, the particles inactivate other phages without the need for an adjuvant to be their immunogen^29^,^30^,^31^,^32^. Our results suggest that the filamentous-phage-display system offers a great promise for developing effective fungal vaccines directed against specific epitopes. In order to develop safe and effective vaccines, further efforts should focus on optimizing the phage, immunization routes, and immune response.

Alba-Fierro *et al*. demonstrated that preimmunization of BALB/c mice with gp60 by intramuscular injection resulted in a downregulatory effect on the immune system^15^. This should be considered in interpreting the immune response of mice, including the concentration, virulence of the inoculum, and the route of infection. It has been suggested that the strength of TCR stimulation critically influences Th1/Th12/Th17 cell differentiation through a mechanism requiring very strong antigen stimulation^19^, Zhong *et al*. reported that peritoneal B1 cells preferentially induce Th1/Th17 cell differentiation *in vitro* due to their greater antigen presentation capacity^33^. Verdan *et al*. demonstrated that high doses of live *S. schenckii* and its exoantigen could activate bone marrow-derived dendritic cells, enabling them to trigger T cell responses *in vitro*^20^. We are therefore led to believe that the intraperitoneal route of infection used in our model may have contributed to the skewed Th1 and Th17 cell development. Phages displaying epitope vaccine could be targeted to antigen-presenting dendritic cells, further stimulating the immunity without adjuvant^34^. The specific immune response, the ability to recruit helper T-cells, and the lack of need for an external adjuvant suggest that phages are a suitable platform for epitope vaccine design. However, the mechanistic basis for phage responses is currently unknown.

In conclusion, we further confirmed that the cellular immune response contributes strongly to host defense against *S. globosa* infection, and antibodies can abrogate establishment and disease development by themselves. The nature of the mechanisms underlying the resolution of infection in mice treated with recombinant phage is a complex issue. Nevertheless, it can be hypothesized that phage-KR can elicit protective antibodies, which could increase the cell-mediated immune response and antibody production. Therefore, phage-KR is a new and safe strategy for the treatment of sporotrichosis.

## Materials and Methods

### Animals

BALB/c mice (6- to 10-week-old mice weighing 20–25 g) were obtained from Beijing HuaFuKang Biological Technology Co. Ltd (Beijing, China). The mice were maintained in an animal facility under specific pathogen-free conditions. The study was approved by the Institutional Ethics Committee for Animal Use in Research and was conducted in accordance with the Animal Care guidelines of the Jilin University.

### Strain, culture conditions, and mouse model

*S. globosa* was isolated from patients diagnosed with invasive sporotrichosis. Molecular identification was used to identify the *S. globosa* isolates. The isolates were maintained in a regular passage and grew on Sabouraud’s dextrose agar tubes at 25 °C. Mycelial-to-yeast phase conversion was accomplished according to the method described by Ferreira *et al*.^19^. Briefly, the isolates were grown on Sabouraud’s dextrose agar slants for 7 days. The fungal colonies were then transferred to brain heart infusion (BHI) broth and incubated in a rotary shaker (150 rpm) at 37 °C for 7 days. The conidia obtained from the cultures were suspended in sterile distilled water and diluted to 1 × 10^8^ or 1 × 10^9^ cells/mL, as determined with a hemocytometer. The mouse model of disseminated *S. globosa* was constructed with intravenous injection of 0.2 mL (2 × 10^7^ cells) of *Sporothrix schenckii* suspension. The yeast cells (10^9^ cells/mL) were killed by heating the cells at 60 °C for 2 h, and heat-killed *S. globosa (*HK-SG) cells were then stored at 4 °C.

### Construction of recombinant phage vector

The phage vector *fuse-55* was propagated in *E. coli* strain TG1, and the wild-type phage used in this procedure was stored in our laboratory. The construction of recombinant phage vector *fuse-55* and the production of recombinant phage were performed as described previously^35^,^36^. Briefly, synthesized complementary oligonucleotides encoding the peptide KR were ligated into the *BglI* -digested phage vector *fuse-55*. E*. coli* TG1 transformed by the recombinant phage vector inserted with the peptide was cultured in Luria-Bertani (LB) liquid medium supplemented with ampicillin. Then, the cultured supernatants containing the phage particles were pooled and purified in polyethylene glycol-6000 (PEG-6000; final concentration, 5%; 0.5 M NaCl) with two consecutive precipitations for 12 h. Then, the phage pellet was resuspended in 2 mL of PBS. The concentration of phage particles was estimated by spectrophotometry (OD of 1.0 at 270 nm was assumed to correspond to a concentration of 0.26 μg/μL).

### SDS-PAGE and Western blotting

#### SDS-PAGE

Expression of the KR peptide generated by the recombinant phage was analyzed using sodium dodecyl sulfate-polyacrylamide gel electrophoresis (SDS-PAGE). The phage samples were boiled for 10 min in an equal volume of 2 × sample loading buffer (4% SDS, 20% glycerol, 100 mM Tris–HCl [pH 8.3], and 0.02% bromphenol blue). Then, the proteins were electrophoresed. The protein bands were revealed using silver staining based on the method described by Schagger *et al*.^37^.

#### Western blotting

The serum obtained from the mouse model with disseminated sporotrichosis contained antibodies against 70 kDa protein of *S. globosa* or control individuals^10^. The protein extracted from the recombinant phage was denatured, electrophoresed, and transblotted onto a nitrocellulose membrane in Tris/glycine buffer. The buffer was blocked overnight at 4 °C in Tris-buffered saline with Tween (TBS-T) containing 5% (w/v) nonfat milk. After washing four times with TBS-T, the nitrocellulose membrane was incubated with serum diluted 1:80 in TBST with 5% nonfat milk at 37 °C for l h. After additional washing, the membrane was incubated with peroxidase-conjugated goat anti-mouse IgG (Vector Laboratories Inc., USA) at 37 °C for 1 h, followed by staining with the chromogen 3-amino-9-ethylcarbozole (AEC).

#### Immunofluorescence

A total of 2 × 10^7^ sporophores were seeded into six-well tissue culture plates and cultured in RPMI-1640 medium for 4 h. After washing slightly with PBS once, the cells were incubated with sera containing antibodies against recombinant phage displaying the peptide kpvqhalltplgldr (RP) or wild-type phage (WP) for 2 h on ice. Adherent cells were fixed with 4% paraformaldehyde for 20 min and then washed three times with PBS. The fixed cells were permeabilized with 0.5% triton X-100 and washed with TBST for three times. After the cells were blocked with 3% bovine serum albumin (BSA) at 37 °C, they were incubated with Cy3-conjugated goat-anti-mouse IgG (1:2000; Abbkine, USA) for 45 min at 37 °C and stained with 4′,6-diamidino-2-phenylindole (DAPI) for 10 min. After washing thrice with PBS with 0.1% Tween-20 (PBST), the cells were mounted and observed with a laser scanning confocal microscope.

#### Immunization schedule

BALB/c mice were divided into four groups. The mice were immunized intraperitoneally at weekly intervals for a total of four times with the following: (i) 25 μg phage-KR in 100 μL of PBS (RP group), (ii) 25 μg wild-type phage particles in 100 μL of PBS (Mock group), (iii) 10^8^ HK-SG in 100 μL of PBS (HK-SG group), or (iv) PBS alone (PBS group; negative control).

### Systemic infection with S. globosa and assessment of protection

The mice were immunized on days 0, 7, 14, and 21 as described above. Then, mice (n = 5, in each group) were intravenously injected via the lateral tail vein (0.2 mL, 2 × 10^7^ cells/mouse) on day 28. Each mouse of the control group received 0.2 mL of PBS alone. In order to determine the colony-forming units (CFU) of *S. globosa* per gram of tissue, the kidneys and livers were aseptically excised from mice (n = 5 per group) 7 days after infection. The kidneys and livers were weighed and homogenized in 3 mL of sterile saline in glass tissue homogenizers. The tissue homogenate was diluted in saline and plated on Sabouraud’s dextrose agar. For histopathological analysis, the tissue samples kidneys and livers were excised and fixed immediately in 10% (v/v) formalin. Then, 3- to 5-μm-sections of paraffin-embedded tissues were stained with hematoxylin and eosin (H&E). In order to determine the survival time of each group (n = 10 per group), the mice in the infection group (1 × 10^8^ cells/mouse) were followed up for 2 weeks, and their survival was evaluated after challenge with live *S. globosa*. The number of dead mice in each group was recorded for two weeks, and from that time on, no more mice died, so the infection was followed for only two weeks.

### Flow cytometry

We collected 50 μL of blood samples using a tube containing sodium heparin. The following Anti-mouse monoclonal antibodies were used to assess frequencies of Th1 (CD45^+^CD3^+^CD4^+^IFN-γ) and Th17 (CD45^+^CD3^+^CD4^+^IL-17^+^) cells: fluorescein isothiocyanate-conjugated anti-IFN-γ, phycoerythrin (PE)-conjugated anti-IL-17, PE-conjugated Cy7-anti-CD45, allophycocyanin (APC)-conjugated anti-CD3, and APC-conjugated Cy7-anti-CD4). The cells were stained for extracellular markers, and fixed and permeabilized with an intracellular fixation and permeabilization buffer set (eBioscience). Finally, the cells were stained to assess the levels of IL-17 and IFN-γ. All fluorophore-conjugated Abs and the corresponding isotypes were purchased from BD Biosciences and eBioscience. The samples were analyzed using BD LSR II and BD FACSDiva software. At least 30,000 events were effectively included in each analysis.

### Toxicity study

Toxicity was assessed 1 day after each immunization. For hematological parameters (red and white blood cell count, and platelet count), blood samples we collected via the lateral tail vein with a tube containing heparin sodium, and they were counted on the Sysmex XE-5000 (Sysmex, Kobe, Japan). To determine serum clinical biochemistry parameters, 50 μL blood samples were collected via the lateral tail vein and transferred to a 0.5-mL centrifugation tube, allowed to clot, and then centrifuged at 4,500× *g* for 5 min. The serum was collected and stored. Serum clinical biochemistry parameters, including aspartate aminotransferase, alanine aminotransferase, alkaline phosphatase, glucose, urea, creatinine, total protein, and albumin, were determined using commercially available kits (Sigma-Aldrich, USA).

### Statistical analysis

Differences in the survival rates were examined using the log-rank test. Data were statistically analyzed using analysis of variance (ANOVA). Statistical significance was set at a *p* value of <0.05.

## Additional Information

**How to cite this article**: Chen, F. *et al*. Recombinant Phage Elicits Protective Immune Response against Systemic S. globosa Infection in Mouse Model. *Sci. Rep.* **7**, 42024; doi: 10.1038/srep42024 (2017).

**Publisher's note:** Springer Nature remains neutral with regard to jurisdictional claims in published maps and institutional affiliations.

## Supplementary Material

Supplementary Information
