# Supplementary material for: Recombinant Phage Elicits Protective Immune Response against Systemic S. globosa Infection in Mouse Model
Source: Sci Rep. 2017 Feb 6;7:42024. doi: 10.1038/srep42024 (PMC5292741; doi:10.1038/srep42024)
Supplement: Supplementary Information [file srep42024-s1.pdf]

Supplementary information

Recombinant Phage Elicits Protective Immune Response against Systemic *S. globosa* Infection in Mouse Model

Feng Chen[1], Rihua Jiang[1, *], Yicun Wang[2], Mingji Zhu[1], Xu Zhang[3], Shuai Dong[4], Hongxi Shi[4], Li Wang[4, *]

[1]Dermatology Department, China-Japan Union Hospital of Jilin University,126Xiantai Street, Changchun, Jilin Province 130033, People's Republic of China

[2]Jilin Provincial Key Laboratory on Molecular and Chemical Genetic, the Second Hospital of Jilin University, 218 Ziqiang Street, Changchun, Jilin Province 130041, People's Republic of China

[3]Department of Hang Surgery, Second Hospital of Qinhuangdao, Qinhuangdao, Hebei 066600, People's Republic of China.

[4]Institute of Cytology and Genetics, School of Life Sciences, Northeast Normal University, 5268 Renmin Street, Changchun, Jilin Province 130024, People's Republic of China

***Corresponding authors:** jrh1963@163.com; wanglee57@163.com

Supplementary Figures and legends

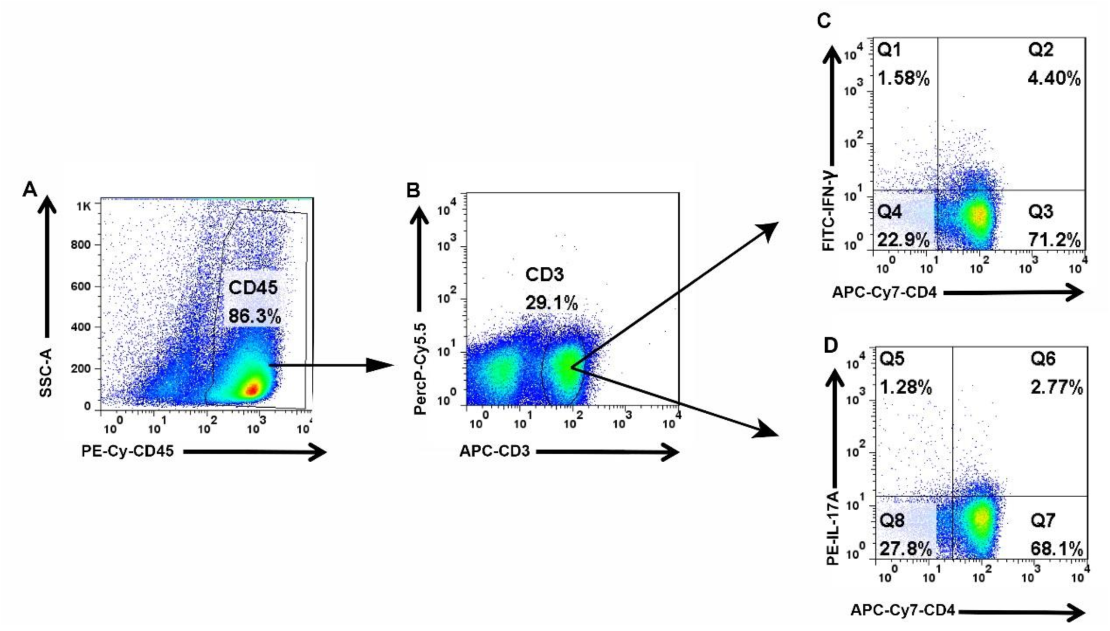

Fig. S1. Gate strategy for Th1 and Th17.

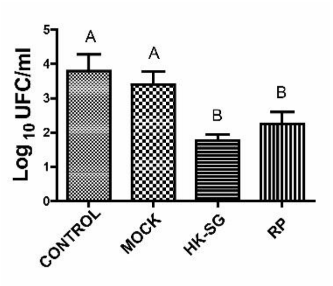

Fig. S2. Log_{10}CFU in livers of mice. To determine whether immunization with hybrid phages could reduce levels of the yeast in organs, mice were killed, and the log_{10}CFU in the livers of mice 7 days after immunization were determined. There were significantly fewer log_{10}CFU in the organs of hybrid phage-immunized mice than in those immunized with phosphate-buffered saline (control group) and wild-type phage (mock group). There was no statistically significant difference in clearance from the liver between the HK-SG-immunized and recombinant phage-immunized groups. Values followed by different capital letters differed significantly between the four groups Control, mock, HK-SG, and RP. RP: recombinant phage, MOCK: wild-type phage. HK-SG: heat-killed *S. globosa*.

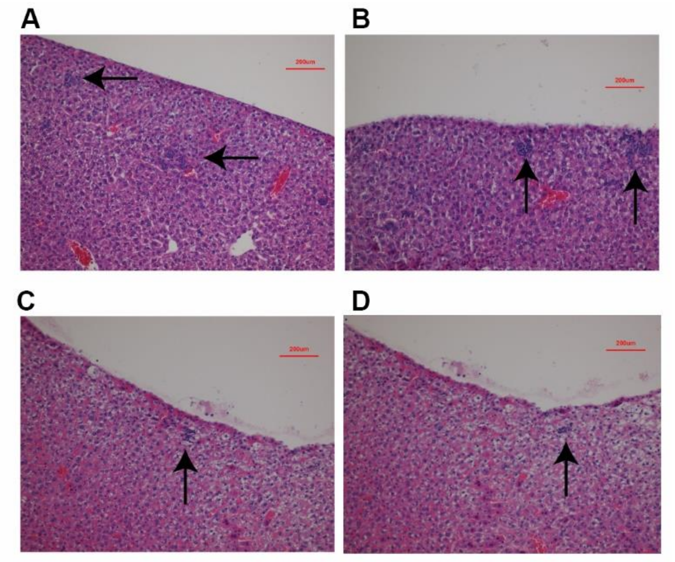

Fig. S3. Representative micrographs of hematoxylin and eosin (HE)-stained livers of *S. globosa*-infected mice. Livers were removed from mice given intraperitoneal injections of PBS (100 μL/mouse), recombinant phage (25 μg/mouse), heat-killed *S. globosa* (10^8 yeast cells/mouse), or wild-type phage (25 μg/mouse) four times at weekly intervals, and then intravenously infected with 2×10^7 *S. globosa* cells. (A) Livers from a mouse injected with PBS. Magnification, 200×. (B) Livers from a mouse immunized with wild-type phage. Magnification, 200×. (C) Liver from a mouse immunized heat-killed *S. globosa* (HK-SG). Magnification, 200×. (D) Liver from a mouse immunized with recombinant phage. Magnification, 200×. Photomicrographs (A) and (B) show a greater number of inflammatory cells in the livers than group HK-SG (C) and group RP (D). The arrows indicate infiltration of inflammatory cells.

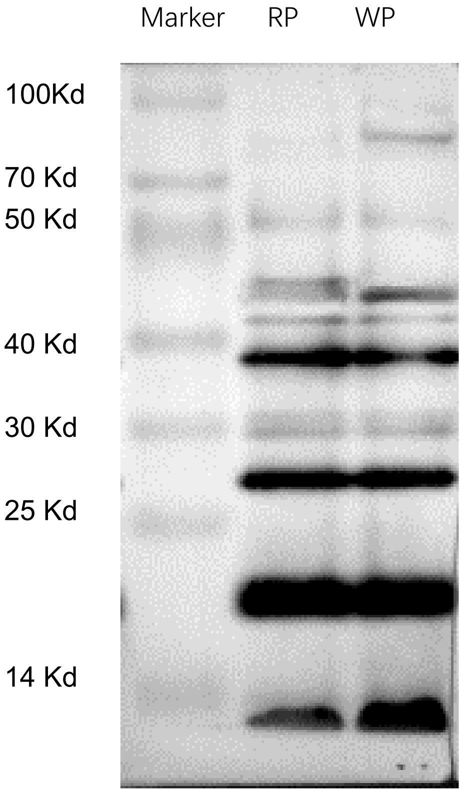

Full-length gels of Fig. 1A RP: recombinant phage, WP: wild-type phage.

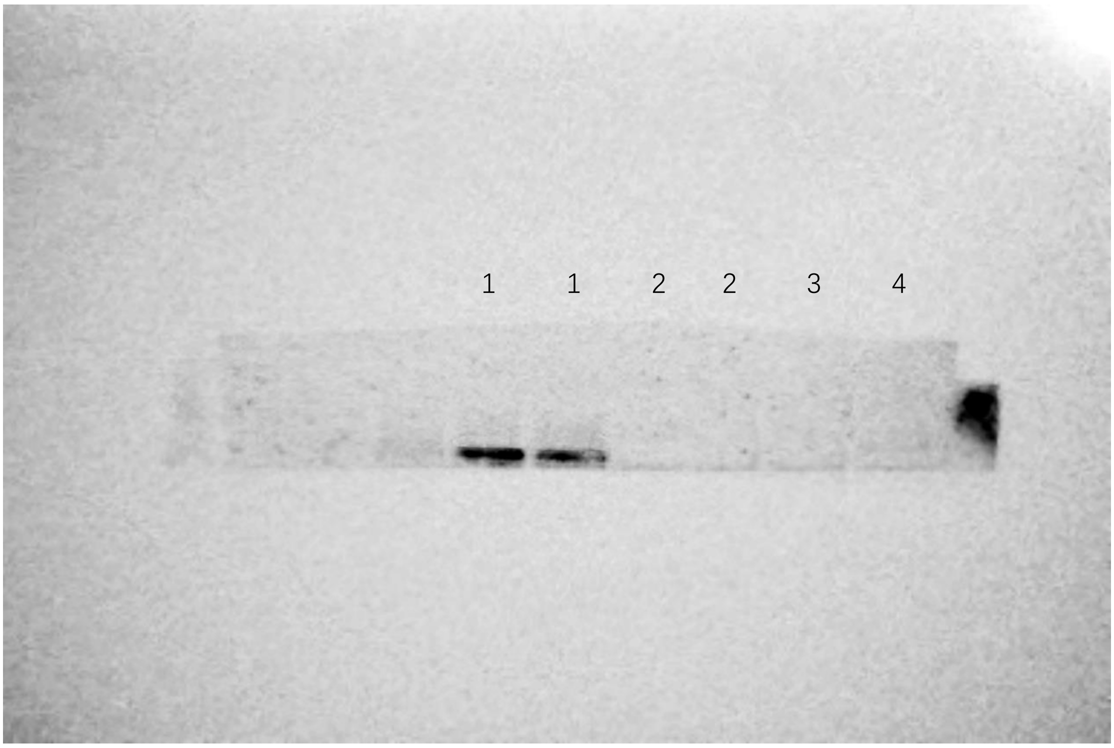

Full-length Blots of Fig. 1B Lanes 1, recombinant phage was probed with infected mouse serum; Lanes 2, wild-type phage was probed with infected mouse serum. Lanes 3, recombinant phage was probed with normal serum. Lanes 4, wild-type phage was probed with normal serum.

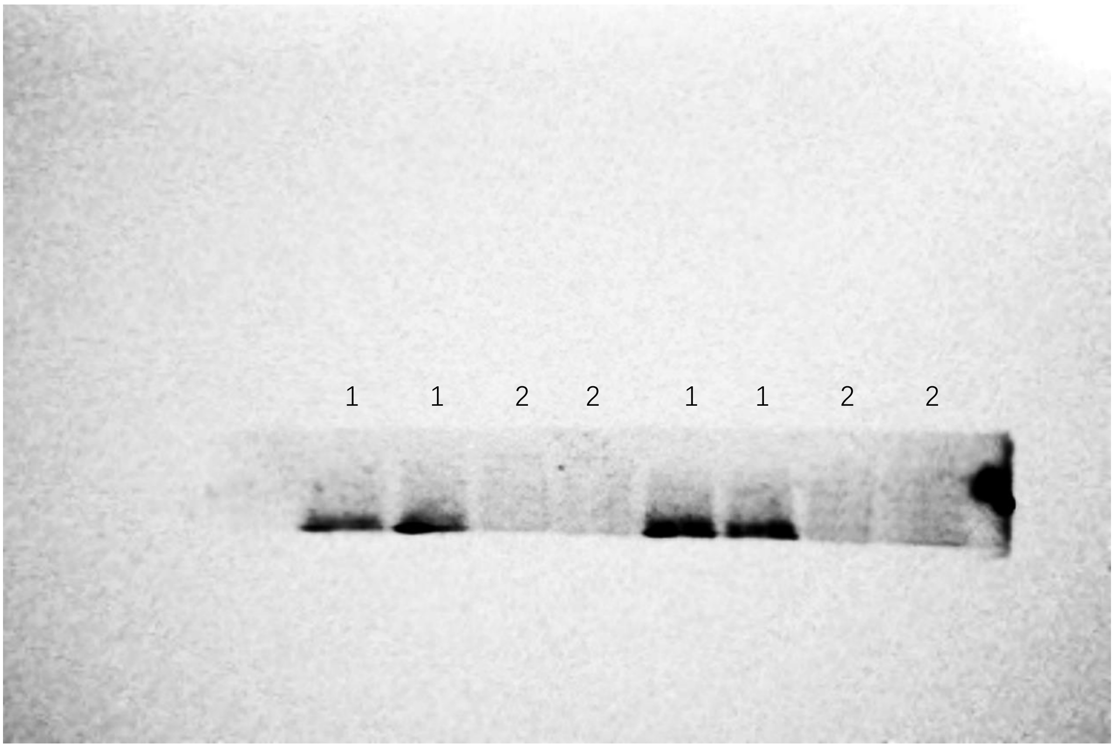

Full-length Blots of Fig. 2 Western blot analysis of sera from recombinant phage-immunized mice with recombinant phage (lane 1), wild-type phage (lane 2), Lanes 1, recombinant phage; Lanes 2, wild-type phage.
